# Comprehensive Evaluation of the Postharvest Antioxidant Capacity of Majiayou Pomelo Harvested at Different Maturities Based on PCA

**DOI:** 10.3390/antiox8050136

**Published:** 2019-05-17

**Authors:** Zhengpeng Nie, Chunpeng Wan, Chuying Chen, Jinyin Chen

**Affiliations:** 1 Collaborative Innovation Center of Post-harvest Key Technology and Quality Safety of Fruits and Vegetables in Jiangxi Province, Jiangxi Key Laboratory for Postharvest Technology and Non-destructive Testing of Fruits & Vegetables, Jiangxi Agricultural University, Nanchang 330045, China; nzp2018@126.com (Z.N.); chunpengwan@jxau.edu.cn (C.W.); 2 Pingxiang University, Pingxiang Jiangxi 337055, China

**Keywords:** Majiayou pomelo (MP), postharvest, antioxidant capacity, harvesting periods, PCA

## Abstract

Majiayou pomelo (*Citrus grandis* L. Osbeck, MP) is a famous local red pulp pomelo from the Jiangxi province in China that is rich in natural active substances. In order to investigate the postharvest antioxidant capacities of MP pulp and determine the optimal harvesting time, fruits that were harvested at three different maturities (185, 200, and 215 days after full bloom) were observed for 180 days of preservation at ambient temperature. An abundance of ascorbic acid and lycopene in the MP pulp was found during storage, and in Harvest I, these substances were significantly higher than in Harvest II and Harvest III fruit (*p* < 0.05). The activity of ascorbate peroxidase (APX), peroxidase (POD), and catalases (CAT) in Harvest I and Harvest II were far higher after 90 days. The radical scavenging ability of 2,2-diphenyl-1-picrylhydrazyl (DPPH) radical, superoxide anion radical (O_2_^−^•), and hydroxyl radical (•OH) in Harvest I and Harvest II were higher. There was a significantly positive correlation (*p* < 0.01) between the antioxidant components (ascorbic acid, lycopene, carotenoids, total phenols, and total flavonoids), enzyme activity, and radical scavenging ability. The comprehensive scores determined by principal component analysis (PCA) in Harvest I and II were higher than those in Harvest III. Therefore, the optimal harvesting period of MP for each year is determined to be early November. The study provides a theoretical basis for the maintenance of the postharvest fruit value and the regulation of fruit functional components.

## 1. Introduction

Majiayou pomelo (*Citrus grandis* L. Osbeck, MP) is a fruit variety that resulted from the breeding of the local pomelo population in Majia Natural village, Danan town, Guangfeng district of Jiangxi province, China, which originated in the Chenghua period of the Ming dynasty (A.D. 1465) and has been cultivated for centuries [1]. In addition, it is listed in the national geographic indication for the protection of products and has been awarded the title of “China’s famous fruit”, resulting in the creation of the top 50 fruit companies in China. It is also the first health food that has passed clinical verification for the treatment of diabetes in China [2]. The MP industry has developed rapidly. The planting area of MP in Shangrao city reached nearly 1.4 thousand hectares (ha) by the end of 2016. The net benefit reached 200 thousand Yuan per ha, which has boosted local economic development [2]. However, the industry now is facing with fruits quality deterioration for excessive pursuit of yield and neglect of management of the harvesting process.

Pomelo fruit is popular among consumers due to its large size, high nutritional value, unique fragrance, and storability [3]. Many researchers have carried out studies on the utilization of pomelo peel [4], but relatively few studies have focused on the bioactive substances in the fruit pulp, which contains phenols, pigments, limonoids, and polysaccharides, providing free radical scavenging, antioxidant, cardioprotective, and anticancer properties [5,6,7]. As the characteristic red pulp pomelo variety, MP has attractive color and taste, and its pulp contains high levels of ascorbic acid, lycopene, beta-carotene, and phenols [8]. The majority of studies on MP have only focused on cultivation management, and the changes of the fruit’s functional components and antioxidant activity during postharvest storage have not yet been explored.

During storage, various reactive oxygen species (ROS) can also be cleared by ROS scavenging systems, such as endogenous antioxidant substances and enzyme systems [9]. Ascorbate peroxidase (APX), peroxidase (POD), superoxide dismutase (SOD), and catalase (CAT) are present as ROS scavengers. SOD turns O_2_^−^• into H_2_O_2_, POD and CAT eliminate H_2_O_2_, and APX removes the H_2_O_2_ by using ascorbic acid as an electron donor. These antioxidant enzymes can scavenge free radicals, reduce the level of membrane lipid peroxidation and the degree of membrane damage, as well as maintain the dynamic balance of the cellular systems [9].

Fruit storability is closely related to harvest time, and different harvest times have different effects on the functional components and antioxidant activity of the fruit [10,11,12]. Fruit harvested too late usually experiences severe tissue aging, accelerated water loss [13], degradation of nutrient components and bioactive substances, and a decline in free radical scavenging capacity [14]. Thus, the determination of optimal harvesting periods will provide a theoretical basis for reducing the postharvest loss of fruit and guide fruit farmers toward producing and harvesting in a scientific way.

Generally, the antioxidant performance of fruits can be reflected by different indicators, but it is difficult to comprehensively analyze the influence of these dispersed indicators. Principal component analysis (PCA) is an analytical method that condenses many variables into a few comprehensive variables, combined with Pearson correlation analysis, which explains the correlation between different variables. Both of these analytical techniques can comprehensively analyze the influence of harvesting maturity on postharvest antioxidant capacity [15]. This method has already been widely applied in the quality evaluation of grapes [16], peaches [17], kiwifruit [18], pomelos [19], and many other fruits.

In this study, the antioxidant capacity of MP pulp, stored at ambient temperature, was comprehensively experimentally evaluated by the determination of the main antioxidant components, the enzyme activity of POD, CAT, APX, and SOD, and the scavenging ability via 2,2-diphenyl-1-picrylhydrazyl (DPPH), •OH, O_2_^−^• and ferric reducing antioxidant power (FRAP) assays. Combined with PCA and Pearson’s correlation analysis, we determined the optimal harvesting period and explored the changes of the functional components during postharvest storage, which provides a basis for the improvement of MP as a health food and reinforces the economic benefits of the MP industry.

## 2. Materials and Methods

### 2.1. Plant Material

MP fruits, from 10-year-old trees grafted on *Fructus aurantii* rootstock, were used. Plants were grown at the base of Majiashan mountain (28.56° N, 117.99° E; 60 m above sea level), at the MP orchard of Jiangxi Guangfeng Xiafeng Green Agriculture Development Co., Ltd., (Shangrao, Jiangxi, China) with a subtropical monsoon humid climate. The management of trees followed local standard agronomic measures, the spacing in the rows and between rows were 3 × 4 m, and the fruits were all covered with paper bags.

### 2.2. Experiment Design and Storage Conditions

According to the local harvesting time of each year, we set three different harvesting maturities for 2017, which were harvested at 185 days after full bloom (October 30th; Harvest I), 200 days after full bloom (November 14th; Harvest II) and 215 days after full bloom (November 29th; Harvest III). For scientific picking, we excluded the marginal trees in the orchard and randomly selected 20 trees, with the same growth and the same slope, for further research. At the time of picking, fruits with the same size, color, and maturity, and which were disease-free. Fruit in the middle part of the tree were picked (except for bore fruits), and at least 15 fruits were picked from each tree. Then, they were packed in plastic woven bags and were transported back to our laboratory. After four days of pre-storage under natural conditions, the paper bags were removed, and fruits were washed under running water and dried naturally, without any treatment. After each fruit was bagged with a 0.03 mm polyethylene bag, 300 fruits were picked for long-term storage in each harvesting maturity. Next, all of the 900 fruits were stored for 180 days at ambient temperature (85–95%, relative humidity), and the fruit samples were sampled with eight fruits for each time at day 0, day 30, day 60, day 90, day 120, day 150, and day 180. After blending with liquid nitrogen frozen, the pomelo fresh samples were preserved in a −80 °C ultra-low temperature refrigerator for further study, and the samples were prepared in duplicate.

### 2.3. Determination of Antioxidant Components

#### 2.3.1. Ascorbic Acid Assay

The content was acquired by a 2,6-dichlorophenol indophenol (DCIP) titration method [20]. About 4 g of sample powder, ground with liquid nitrogen, was mixed with 50 mL of 2% (*m*/*v*) oxalic acid solution. Then, 10 mL of the solution was transferred to a triangular flask (50 mL), and the DCIP solution that had been calibrated was immediately performed for sample solution titration. The terminal point was recorded, with a reddish appearance at 15 s fadeless. The ascorbic acid content was calculated by the consumed volume of the DCIP solution.

#### 2.3.2. Lycopene and Carotenoids Assay

The extraction method of lycopene referred to the method that was optimized by Xu et al. [21]. Of the pulp sample powder, 1.0 g, ground in liquid nitrogen, was pretreated with 95% ethanol for 30 min, centrifuged at 4000 r/min for precipitation, and petroleum ether (3.5 mL) was added to the precipitation. Then, it was incubated at 30 °C in a thermostatic oscillator, with aluminium-foil paper to avoid light, and extracted for 3.8 h to dissolve it in organic solvent. After extraction, it was kept in the dark for 10 minutes, and the organic phase in the upper layer was the extraction solution. The measurement method of lycopene was described by Martínez-Valverde et al. [22]. The content of lycopene was expressed as μg/g and was calculated by the molar extinction coefficient at a wavelength of 472 nm.

Carotenoids were measured according to the method described previously [23], with few modifications. About 0.5 g of the sample powder was added with the extraction agent (15 mL, hexane:acetone:anhydrous alcohol = 2:1:1, *v*/*v*). Then, was ultrasonically extracted for 30 min, keeping it out of the sun in the meantime. Afterwards, it was centrifuged at 4000 r/min for 10 min to obtain the supernatant. The precipitate was extracted twice with the extraction agent (15 mL) in the same way until it was colorless, combining the supernatant and allowing a constant volume to 45 mL. The absorbance value was determined at a wavelength of 450 nm, with a blank extraction solvent. The content of carotenoids was expressed as μg/g and was calculated by the average extinction coefficient at a wavelength of 450 nm of the standard β-carotenoids.

#### 2.3.3. The Total Phenolic and Total Flavonoid Contents Assay

The total phenolic content was assayed following the Folin–Ciocalteu (FC) method [24], with few modifications. The extraction solution (0.5 mL) was mixed with distilled water (5 mL) and FC reagent (0.5 mL). The mixed solution was set for 5 min after blending, then 1 mL of sodium carbonate solution (10% mass fraction) was added to it, and the absorbance at a wavelength of 725 nm was measured, after incubation in light for 1 h. Gallic acid was used as a standard sample to make the standard curve, and the total phenolic content was expressed as mg/g.

The aluminium nitrate colorimetric method [25] was applied to determine the total flavonoid content, with certain alterations. About 0.3 mL of sodium nitrite (5% mass fraction) was added to the extraction solution (2.4 mL). After blending and incubating it for 6 min, 0.3 mL of aluminium nitrate (10% mass fraction) was added. Then, it was incubated for another 6 min, 4 mL of sodium hydroxide (1 M) was added to it, and the absorbance at a wavelength of 510 nm was measured, after incubation in light for another 15 min. Rutin was used as a standard sample to make the standard curve, and the total flavonoid content was expressed as mg/g.

### 2.4. Enzyme Extraction and Activity Assays

#### 2.4.1. SOD, POD, and CAT Activity Assays

A total of 3.0 g of frozen pulp powder, grinded by liquid nitrogen, was added to the precooling 5.0 mL of phosphate buffer solution (PBS, 50 mM, pH 7.8) and quartz sand (0.2 g) for ice-bath grinding. Later, the homogenate was centrifuged at 16,000 r/min for 20 min at 4 °C, and the supernatant stored at 4 °C was used for the determination of the SOD activity, POD activity and CAT activity. 

The SOD activity was determined by the nitro-blue tetrazolium (NBT) method [26], with some modifications. The reaction mixture was made of 1.5 mL PBS (50 mM, pH 7.8), 0.3 mL ethylene diamine tetraacetic acid disodium (EDTA-Na_2_, 10 mM), 0.3 mL methionine (130 mM), 0.3 mL NBT (750 μM), 0.3 mL riboflavin (40 μM), 0.1 mL of crude enzyme extract and 0.5 mL of distilled water, and the control group received distilled water instead. After adding riboflavin, the control group was immediately placed in the shade, with a cover made of a double layer of black cardboard. Then, the reaction mixture was placed under a 4000 lx fluorescent lamp for 15–20 min at 25–35 °C for the chromogenic reaction. Finally, the absorbance at a wavelength of 560 nm was measured, and one unit of the enzyme activity was equal to the amount of enzyme required to inhibit 50% of the NBT photoreduction reaction. The SOD activity was expressed as U g^−1^ h^−1^.

The POD activity was acquired by the guaiacol method [26]. The reaction solution included 3.0 mL of guaiacol (25 mM), 0.5 mL of crude enzyme extract and 200 μL of hydrogen peroxide (0.5 M). After hydrogen peroxide was added, the solution was quickly mixed to initiate the reaction and start the timer. An absorbance value at a wavelength of 470 nm at 15 s was the initial value, recording the consequent increase of absorbance in 5 min, and distilled water was the reference. The POD activity was expressed as U g^−1^ min^−1^.

The CAT activity was obtained by the ultraviolet colorimetry method [26]. The reaction system consisted of 2.9 mL of hydrogen peroxide (20 mM) and 0.1 mL of crude enzyme extract, and distilled water was used as a control. An absorbance value at a wavelength of 240 nm at 15 s was recorded as the initial value, which was recorded every 30 s, and more than six data points were continuously measured. The CAT activity was expressed as U g^−1^ min^−1^.

#### 2.4.2. APX Activity Assay

The APX activity adopted the ultraviolet colorimetry method described by Amako and colleagues [27]. A weighted amount of 0.5 g of frozen fruit pulp powder was homogenized with 5 mL PBS (0.1 M, pH 7.5), pre-cooled at 4 °C, containing EDTA (0.1 mM), ascorbic acid (1 mM) and polyvinylpyrrolidone (2%, PVPP). The homogenate was centrifuged at 12,000 r/min for 30 min at 4 °C, and the resulting supernatant was used for the APX activity determination. The reaction mixture was made of 2.6 mL PBS (50 mM, pH 7.5), pre-cooled at 4 °C, containing EDTA (0.1 mM) and ascorbic acid (1 mM), crude enzyme extract (0.1 mL) and hydrogen peroxide (2 mM), and distilled water was used as a control. The determination method was consistent with the CAT enzyme. An absorbance value at a wavelength of 290 nm was recorded, and the APX activity was expressed as U g^−1^ min^−1^.

### 2.5. Assessment of Antioxidant Capacity

#### 2.5.1. Antioxidants Extraction

For the extraction of the antioxidants, 0.5 g of the pulp sample powder, grinded by liquid nitrogen, was added with 10 mL methanol. After shaking it well, it was extracted ultrasonically at 50 °C for 30 min and centrifuged at 5000 r/min for 15 min to obtain the supernatant. The precipitate was extracted twice, with a 10 mL extraction agent, in the same way, until it was colorless. Then, the supernatant was combined, allowed a constant volume with methanol, and preserved in −40 °C for the determination of the antioxidant assay.

#### 2.5.2. DPPH Radical Scavenging Assay

The DPPH assay [28] was used to evaluate the total antioxidant activity. DPPH reserve solution (0.2 mM) was prepared with anhydrous ethanol. After shaking it well, the mixed liquor was incubated at ambient temperature under light conditions for 30 min. The absorbance values of A_0_ (2.0 mL DPPH reserve solution and 2.0 mL 70% ethanol), A_1_ (2.0 mL DPPH reserve solution and 2.0 mL extraction solution) and A_2_ (2.0 mL 70% ethanol and 2.0 mL extraction solution) at a wavelength of 517 nm were measured for the DPPH scavenging capacity, which was expressed as follows: scavenging rate (%) = [(A_0_ − A_1_ + A_2_)/A_0_] × 100%.

#### 2.5.3. Superoxide Radical Scavenging Assay

The superoxide anoin radical scavenging capacity was measured by the pyrogallol autoxidation method, with few modifications [29]. About 4.5 mL of Tris-HCl buffer (50 mM, pH 8.2) was mixed with distilled water (3.3 mL) and incubated for 20 min at 25 °C. Then, 0.9 mL of extraction solution and 0.3 mL of catechol solution (3 mM, prepared with 10 mM HCl by 25 °C pre-heat) were quickly added to it. The absorbance of the reaction solution at a wavelength of 320 nm was recorded within 5 min after blending and named V_1_ (absorbance changes after every minute). Following the method, described above, the extracted solution was replaced by distilled water for the reaction, and V_0_ was obtained. The scavenging rate (%) = [1 − V_1_/V_0_] × 100%.

#### 2.5.4. Hydroxyl radicals scavenging assay

The hydroxyl radicals scavenging assay is based on the reaction between salicylic acid and the Fenton process [30]. Of the extract solution, 1.0 mL was added with 1.0 mL of ferrous sulfate solution (9 mM, FeSO_4_·7H_2_O) and 1 mL hydrogen peroxide solution (10 mM), and it was incubated at 37 °C in a water bath for 10 min. Then, 1 mL of salicylic acid solution was added (9 mM), maintaining it at 37 °C for 30 min in a water bath, and the absorbance of the reaction solution at a wavelength of 510 nm was measured, named A_1_. Following the method, described above, the extracted solution was replaced by distilled water for the reaction, and A_0_ was obtained. The scavenging rate (%) = [1 − A_1_/A_0_] × 100%.

#### 2.5.5. Ferric-Reducing Antioxidant Power Assay

The FRAP was assayed following a previously described method [31], where 2.5 mL of the extraction solution was mixed with 2.5 mL PBS (0.2 mM, pH 6.6) and 2.5 mL potassium ferricyanide solution (1% mass fraction), and it was incubated at 50 °C for 20 min. After cooling rapidly, 2.5 mL of trichloroacetic acid solution (10% mass fraction) was added to it. Then, the mixture was centrifuged at 3000 r/min for 10 min to obtain the supernatant. The absorbance at a wavelength of 700 nm of 2.5 mL supernatant, 2.5 mL distilled water and 0.5 mL ferric trichloride solution (0.1% mass fraction) was measured, and this represents the ferric reducing ability. In addition, the higher the absorbance value, the stronger the reducing ability.

### 2.6. Statistical Analysis

Data statistics and drawing were performed by Excel 2013 software and Origin 8.0 software, respectively. All experimental results were expressed as the mean value and standard error of more than three repetitions. IBM SPSS 20.0 software (IBM-SPSS, Armonk, NY, USA) was used for the analysis of variance (ANOVA), Pearson correlation analysis and PCA. The significant difference level is set at *p* < 0.05 and *p* < 0.01.

## 3. Results

### 3.1. Changes of the Antioxidant Components of MP

**Figure 1:**
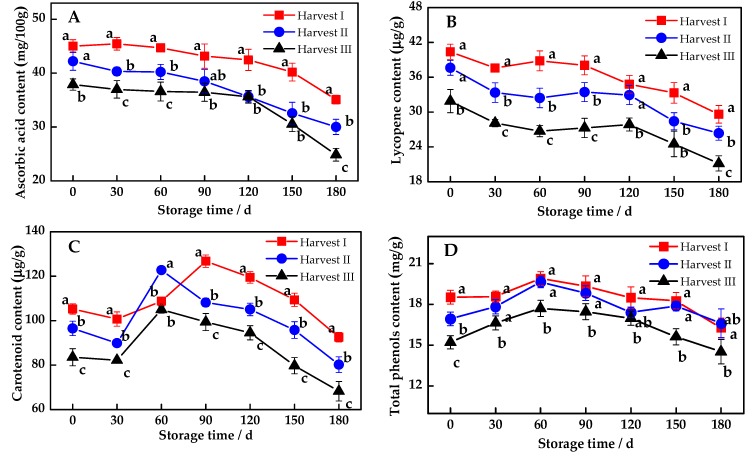

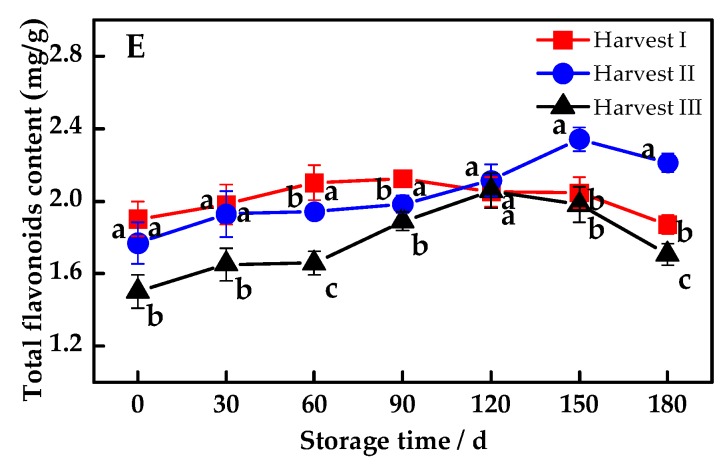
Dynamic changes for the antioxidant substance of Majiayou pomelo (MP) during storage at ambient temperature. (**A**) Changes for the ascorbic acid content; (**B**) Changes for the lycopene content; (**C**) Changes for the carotenoid content; (**D**) Changes for the total phenols content; (**E**) Changes for the total flavonoids content. The different letters for the same storage time indicates a significant difference (*p* < 0.05).

Ascorbic acid is an essential nutrient substance of the citrus variety, which has tremendous effects on fruit quality, disease resistance and anti-aging ability. The ascorbic acid in pomelo fruits decreased continuously along with the storage time (Figure 1A). While the ascorbic acid of Harvest II and Harvest III decreased rapidly after 90 days of storage, that of Harvest I only dropped by 22.04% during the entire storage and always maintained a high level, which was significantly higher than the other two (*p* < 0.05).

Lycopene and carotenoids are functional natural pigments of red fresh pomelo. Similar to the changes in ascorbic acid, lycopene also declined as the storage period was prolonged (Figure 1B). While carotenoids and total phenols first increased and then decreased (Figure 1C,D), the total flavonoids (Figure 1E) content had a slight fluctuation in its contents. Comparing the harvesting periods, the lycopene of Harvest I was always higher than that of Harvest II and Harvest III, and there existed a significant difference between Harvest I and Harvest III (*p* < 0.05). The carotenoids of Harvest I and Harvest II were significantly higher than those of Harvest III after 60 days of storage (*p* < 0.05), and the total phenols of Harvest I were significantly higher than those of Harvest III. In addition to the flavonoids, Harvest I and Harvest II can maintain these functional components at a high level and maintain a strong biological activity and better defense against fruit oxidative stress.

### 3.2. Changes of Antioxidant Enzyme of MP

**Figure 2:**
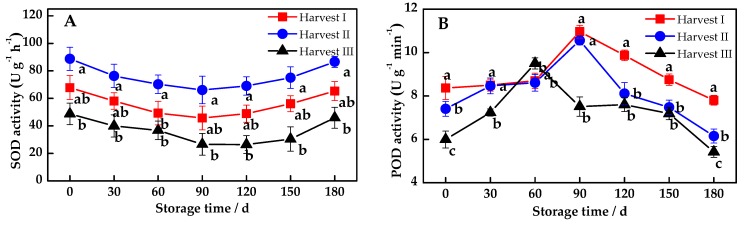

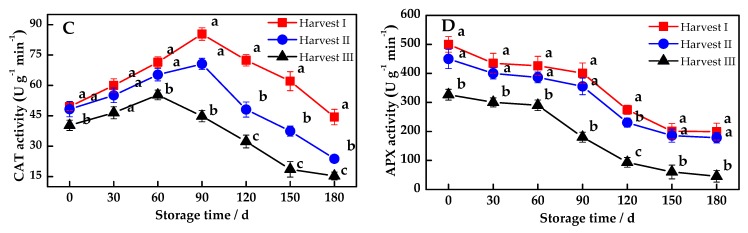
Dynamic changes for the antioxidant enzymes activities of Majiayou pomelo (MP) during storage at ambient temperature. (**A**) Changes for the superoxide dismutase (SOD) activity; (**B**) Changes for the peroxidase (POD) activity; (**C**) Changes for the catalase (CAT) activity; (**D**) Changes for the ascorbate peroxidase (APX) activity. The different letters for the same storage time indicates a significant difference (*p* < 0.05).

The ROS scavenging enzyme system consisted of SOD, POD, CAT and APX, and is capable of reducing the accumulation of free radicals to alleviate ROS-mediated damage. The SOD activity rose slightly after falling a little, and there was little fluctuation (Figure 2A). Meanwhile, its activity in Harvest II was significantly higher than in Harvest III (*p* < 0.05), and there was no significant difference between Harvest I and Harvest II. The POD activity (Figure 2B) had almost the same variation trend as the CAT activity (Figure 2C). The POD and CAT activity of Harvest III reached a peak at 60 days of storage, while Harvest I and Harvest II maximized at 90 days of storage. After 90 days of storage, Harvest I and Harvest II were much higher than Harvest III, and there was a significant difference between Harvest I and Harvest III (*p* < 0.05). Furthermore, Harvest I and Harvest II could defer the advent of the enzyme activity peak in POD and CAT, and both maintained a relatively high enzyme activity in the middle and later periods of the storage. APX is a key enzyme in the ascorbic acid–glutathione cycle, and its variation was due to ascorbic acid. Obviously, APX presented a declining trend as a whole, and the decline rate was faster after 90 days of storage (Figure 2D). Besides, the APX activity of Harvest I and Harvest II were significantly higher than that of Harvest III (*p* < 0.05), but there was no significant difference between Harvest I and Harvest II. Thus, Harvest I and Harvest II could delay the decline of the APX activity to a certain extent, promoting the resistance to stress for pomelo fruit during storage.

### 3.3. Changes of the Antioxidant Capacity of MP

**Figure 3:**
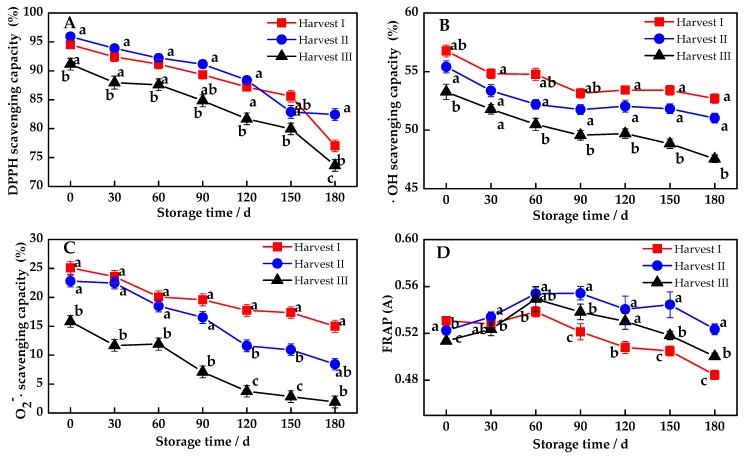
Dynamic changes for the antioxidant capacity of Majiayou pomelo (MP) during storage at ambient temperature. (**A**) Changes for the 2,2-diphenyl-1-picrylhydrazyl (DPPH) scavenging capacity; (**B**) Changes for the hydroxyl radical (•OH) scavenging capacity; (**C**) Changes for the superoxide anions radical (O_2_^−^•) scavenging capacity; (**D**) Changes for the ferric reducing antioxidant power (FRAP). The different letters for the same storage time indicates a significant difference (*p* < 0.05).

Pomelo fruit contains phenols and other antioxidants, and thus its antioxidant capacity is also of great concern. In this experiment, the DPPH radical scavenging method, which is widely applied and highly sensitive, combined with the hydroxyl radical scavenging method, superoxide anions radical scavenging method and FRAP assay, was used to determine the pomelo pulp’s antioxidant capacity. The scavenging rate of DPPH, •OH and O_2_^−^• decreased, following storage time, as shown in Figure 3A–C. Moreover, there was no significant difference between Harvest I and Harvest II in terms of the scavenging capacity of DPPH and •OH, but the scavenging capacity was significantly higher for these two harvesting times than for Harvest III (*p* < 0.01). At the same time, the superoxide radical scavenging ability of Harvest III was at its lowest level among the three harvesting times, and it was significantly lower than that of Harvest I and Harvest II (*p* < 0.05). As for FRAP, it showed a tendency of increasing first and then decreasing during storage (Figure 3D), and the reducing ability in Harvest II was significantly higher than in Harvest I and Harvest III, after 60 days of storage (*p* < 0.05), which indicated that Harvest II could effectively slow its descent, compared with the others. To sum up, Harvest I and Harvest II had a stronger antioxidant capacity than Harvest III.

### 3.4. Correlation Analysis of Different Indexes of MP

**Figure 4:**
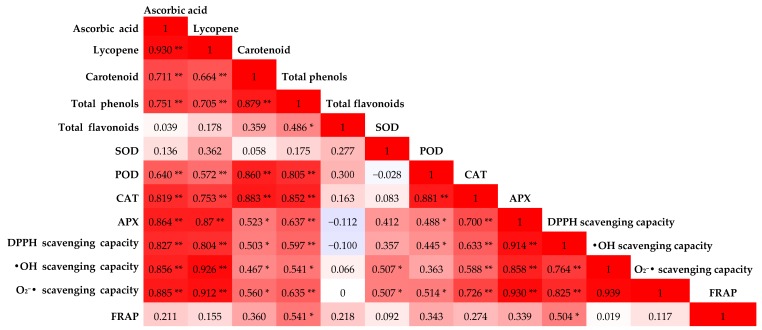
Correlation matrix based on Pearson’s correlation coefficient between the different antioxidant substances, antioxidant enzymes and antioxidant capacities. The color intensity and numbers are proportional to the correlation coefficients. Positive correlations are shown in red, and negative correlations, in blue. *,** means a significance of *p* = 0.05 and *p* = 0.01, respectively.

Pearson correlation analysis can reveal the degree of correlation between two parameters. Antioxidant components are consumed continuously under ambient temperature storage, and the antioxidant enzyme activity and free radical scavenging ability, whose internal relationships were expressed in the form of correlation coefficient, are weakened accordingly. Obviously, there were multiple sets of significant correlations among different indexes (*p* = 0.05 or *p* = 0.01) during the storage of MP (Figure 4). As for the antioxidant components, there were significant positive correlations among ascorbic acid, lycopene, carotenoid and total phenol (*p* = 0.01). The correlation between ascorbic acid and lycopene (*r* = 0.930, *p* = 0.01), total phenol and carotenoids (*r* = 0.879, *p* = 0.01) was higher. At the same time, the correlation between ascorbic acid and lycopene (*r* = 0.930, *p* = 0.01), total phenol and carotenoids (*r* = 0.879, *p* = 0.01) was quite high. However, the correlation coefficient of the total flavonoids and other antioxidant components (ascorbic acid, lycopene, carotenoids and total phenols) was relatively low and was considered a low correlation. This was the same for all of the antioxidant components and SOD and FRAP. These results indicated that the total flavonoids were probably not the main contributor to the antioxidant capacity of the pomelo fruit during storage, and there were differences among the different antioxidant evaluation systems, like SOD and FRAP. In addition, there were significant or extremely significant correlations among antioxidant components, antioxidant enzymes and free radical scavenging ability. The high correlations were between two antioxidant components (ascorbic acid and lycopene) and APX activity and free radical scavenging ability (DPPH, •OH, O_2_^−^•), the other two (carotenoids and total phenol) and enzyme activity (POD and CAT), whose correlation coefficients were all more than 0.8, and were of a significant correlation (*p* = 0.01). These results indicated that these antioxidant components determined the antioxidant performance of pomelo pulp to a large extent, and the evaluation results were of a high reliability.

### 3.5. PCA on the Postharvest Antioxidant Capacity of MP

According to the preliminary experimental results, we standardized 13 indexes of MP in the postharvest storage process for PCA by using SPSS 20.0 software. Three principal components were extracted, and the cumulative variance contribution rate reached 85.633% (Table 1), which could reflect most information from the original data. According to the load values of each principal component in Table 2, the variance contribution rate of principal component 1 (45.124%) is the highest, and ascorbic acid, lycopene, APX activity, and scavenging capacity of DPPH, •OH and O_2_^−^• all had a bigger loading on it. As for the variance contribution rate of principal component 2, it was 30.256%, and the carotenoids, total phenols, POD activity, CAT activity and FRAP all had a large loading on it. Similarly, the variance contribution rate of principal component 3 was 10.252%, and the total flavonoids and SOD activity had a large load on it. The above three aspects constitute the comprehensive postharvest antioxidant capacity of MP.

In order to further compare the differences of the postharvest antioxidant capacity at each harvesting period, combining the standardized indexes (X_1_–X_13_) and factor load value, we obtained the scores of each principal component, which is shown in the following formula:

*F*_1_ = 0.844*X*_1_ + 0.868*X*_2_ + 0.382*X*_3_ + 0.436*X*_4_ − 0.234*X*_5_ + 0.448*X*_6_ + 0.317*X*_7_ + 0.583*X*_8_ + 0.932*X*_9_ + 0.855*X*_10_ + 0.931*X*_11_ + 0.947*X*_12_ + 0.054*X*_13_

*F*_2_ = 0.458*X*_1_ + 0.380*X*_2_ + 0.858*X*_3_ + 0.856*X*_4_ + 0.562*X*_5_ − 0.153*X*_6_ + 0.858*X*_7_ + 0.737*X*_8_ + 0.245*X*_9_ + 0.270*X*_10_ + 0.118*X*_11_ + 0.229*X*_12_ + 0.539*X*_13_

*F*_3_ = −0.130*X*_1_ + 0.117*X*_2_ − 0.032*X*_3_ + 0.160*X*_4_ + 0.809*X*_5_ + 0.699*X*_6_ − 0.137*X*_7_ − 0.155*X*_8_ + 0.028*X*_9_ + 0.026*X*_10_ + 0.203*X*_11_ + 0.123*X*_12_ + 0.179*X*_13_

Then, the score of each principal component was multiplied by the contribution rate of variance, that is, the weight of each principal component, and we obtained the comprehensive model of the postharvest antioxidant capacity of MP:

*F* = 0.45124*F*_1_ + 0.30256*F*_2_ + 0.10252*F*_3_

**Figure 5:**
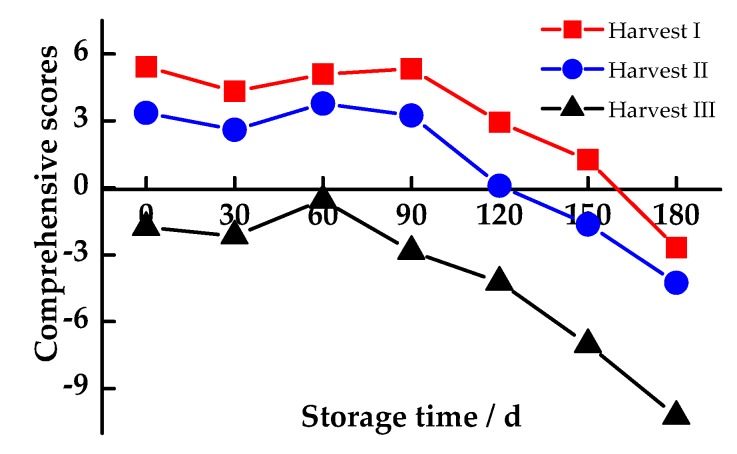
Comprehensive scores of the postharvest antioxidant capacity for Majiayou pomelo (MP) at different harvesting periods during storage at ambient temperature.

Figure 5 shows the comprehensive scores of MP in the postharvest storage at different harvesting periods. Taking the composite score of 0 as the benchmark, a positive value indicates a strong antioxidant capacity, and a higher value indicates a stronger capacity, while a negative value indicates a poor capacity. Obviously, the composite score rose gently, after a slight decline after 30 days. After storage for 90 days, the fall of the comprehensive scores was accelerated, with the worst oxidation resistance. Harvest III was negative during the whole storage period, and Harvest I and Harvest II became negative after storage for 150 days and 120 days, respectively, which was 60 days longer than for Harvest III. Furthermore, the comprehensive scores of Harvest I and Harvest II were much higher than those of Harvest III during the whole storage period. However, the scores of Harvest I and Harvest II were relatively close, so the antioxidant capacity difference is not significant. Thus, considering the comprehensive postharvest antioxidant capacity, Harvest I and Harvest II are significantly better than Harvest III.

## 4. Discussion

Pomelo fruits aging during storage is a relatively complex process that is accompanied by a series of physiological and biochemical changes [32]. Faced with abiotic stresses that lead to fruit tissue disorders in its various functional components, including ascorbic acid, flavonoids, carotenoids and phenolic compounds, occurring at different degrees of synthesis or degradation [33]. What we observed in the storage process of MP at ambient temperature was consistent with previous studies. While most of the antioxidants, like ascorbic acid and lycopene, were constantly and gradually degraded by consumption as respiratory substrates during the aging process of pomelo fruits. The carotenoids and total phenols were increased before storage for 60 days and then declined. Xu and Deng [34] found that carotenoids might continue to be synthesized in the process of postharvest storage, initially leading to an increase in its contents at the early stage of storage. Besides, the total phenolic content is influenced by ascorbic acid, flavonoids and phenolic acids, and the phenolic substances can be released from the proteins and sugars mixtures [35]. It is speculated that the synthesized amount of total phenols in the early stage of storage is higher than the decomposition amount of it, resulting in an increased content. As for the little fluctuation of the total flavonoids content, it was probably due to its low contents in the pomelo pulp [36]. Besides, most of the pigments and phenolic substances reached their peak at 90 days of storage, which was enriched with nutritious compounds that are beneficial for health. Thus, the storage time at ambient temperature should be controlled within 90 days. After that, the fruit pigment will gradually decompose, and the original flavor disappears.

The free radical scavenging ability reflects the antioxidant capacity of fruits to a certain extent, and accordingly, the free radical accumulation causes tissue damage and organ aging, which is thought to be a significant tool for measuring the aging of fruits and vegetables [37]. Generally, the higher the free radical scavenging rate, the lower the degree of aging, and the better the fruit quality. The Pearson’s correlation analysis showed a decrease in the scavenging rate of DPPH, and •OH and O_2_^−^• tended to be synchronized with the consumption of ascorbic acid and lycopene. This indicated that these various substances had a great contribution to the antioxidant capacity of pomelo fruit under storage [38]. When the correlation coefficients of each nutrient component with the scavenging power of •OH and O_2_^−^• were compared, lycopene and ascorbic acid showed a stronger correlation than the rest of the components, followed by carotenoids and total phenols. No significant correlation with flavonoids was seen, which demonstrates that lycopene and ascorbic acid are probably the main contributors to the antioxidant capacity of MP during storage.

In the process of fruit aging, the accumulation of intracellular ROS would continue to increase and enhance the membrane lipid peroxidation, damage the organic structure and finally lead to fruit decay [39]. Fruits being harvested too early or late may affect the levels of ROS scavenging enzymes (POD, SOD, CAT and APX), thus disrupting the cellular homeostasis of redox metabolism [40]. The results showed that the enzyme activity (POD, CAT and APX) of different harvesting periods accelerated the decrease after 90 days of storage. This indicated that the harvesting period had a great influence on the redox metabolism during the long-term storage of pomelo fruits. In addition, the enzyme activities of Harvest I and Harvest II were both higher in the later storage period than in Harvest III, and the former delayed the arrival of the enzyme activity peak. The late harvesting of MP may cause the disturbance of redox metabolism in the later storage period, which is not conducive to the storage of pomelo fruits.

PCA is an authentic tool that simplifies the multiple evaluation indexes and has become one of the main methods for the comprehensive quality evaluation of fruits, vegetables and foods [41,42]. Herein, the PCA was applied to extract 4 principal components from 13 antioxidant indexes, which reflected 85.633% of the information of all indexes. After the main indexes were standardized, the scores of the three principal components were calculated. The product of each score and the variance contribution percentage of the corresponding characteristic value was accumulated to obtain the comprehensive evaluation of the antioxidant capacity for each harvesting period. Obviously, the scores decreased sharply after 90 days of storage, indicating that the optimal storage time of pomelo fruit is 90 days, after the appropriate harvest. When the storage time was too long, the fruit had a poor anti-oxidative and anti-aging ability. In addition, the comprehensive score of Harvest I was always the highest among the three, which was close to Harvest II, with a strong antioxidant performance. Therefore, the periods, i.e., 185 to 200 days after full bloom, should be considered as a suitable harvesting period for MP.

## 5. Conclusions

In this research, Harvest I and Harvest II significantly reduced the loss of functional components, maintained higher antioxidant enzyme activities and kept a stronger ability to scavenge free radicals after 180 days of storing MP at ambient temperature, indicating that it is better to maintain the fruit’s commodity value after harvesting. Besides, the comprehensive scores of Harvest I and Harvest II during storage were far higher than the other, so the recommended harvesting time of MP for each year is determined to be early November, namely, 185 to 200 days after full bloom. This study will not only provide guidance for production practices, but also a baseline reference for future research on the change rules of effective functional components in the postharvest process of MP.

## Figures and Tables

**Table 1:** Eigenvalue and accumulative contribution rate of the postharvest quality evaluation of Majiayou pomelo (MP).

Principal Component	Characteristic Value	Variance Contribution Rate (%)	The Cumulative Variance Contribution (%)
1	5.866	45.124	45.124
2	3.933	30.256	75.381
3	1.333	10.252	85.633

**Table 2:** Rotated component matrix of the principle component analysis.

Standardized Code Name	Indexes	Principal Components		
		1	2	3
X_1_	Ascorbic acid	0.844	0.458	−0.130
X_2_	Lycopene	0.868	0.380	0.117
X_3_	Carotenoid	0.382	0.858	−0.032
X_4_	Total phenols	0.436	0.856	0.160
X_5_	Total flavonoids	−0.234	0.562	0.699
X_6_	SOD	0.448	−0.153	0.809
X_7_	POD	0.317	0.858	−0.137
X_8_	CAT	0.583	0.737	−0.155
X_9_	APX	0.932	0.245	0.028
X_10_	DPPH scavenging capacity	0.855	0.270	0.026
X_11_	•OH scavenging capacity	0.931	0.118	0.203
X_12_	O_2_^−^• scavenging capacity	0.947	0.229	0.123
X_13_	FRAP	0.054	0.539	0.179
